# Diagnostic yield of exome and genome sequencing after non-diagnostic multi-gene panels in patients with single-system diseases

**DOI:** 10.1186/s13023-024-03213-x

**Published:** 2024-05-24

**Authors:** Matheus V. M. B. Wilke, Eric W. Klee, Radhika Dhamija, Fernando C. Fervenza, Brittany Thomas, Nelson Leung, Marie C. Hogan, Megan M. Hager, Kayla J. Kolbert, Jennifer L. Kemppainen, Elle C. Loftus, Katie M. Leitzen, Carolyn R. Vitek, Tammy McAllister, Konstantinos N. Lazaridis, Filippo Pinto e Vairo

**Affiliations:** 1 Center for Individualized Medicine, Mayo Clinic, Rochester, MN USA; 2 Department of Clinical Genomics, Mayo Clinic, Rochester, MN USA; 3 Department of Quantitative Health Sciences, Mayo Clinic, Rochester, MN USA; 4 Division of Nephrology and Hypertension, Mayo Clinic, Rochester, MN USA; 5 Illumina, Inc, San Diego, CA USA; 6 Natera, Austin, TX USA; 7 Division of Gastroenterology & Hepatology, Mayo Clinic, Rochester, MN USA

**Keywords:** Rare diseases, Genetic testing, Diagnostic yield, Gene panels, Exome, Genome

## Abstract

Though next-generation sequencing (NGS) tests like exome sequencing (ES), genome sequencing (GS), and panels derived from exome and genome data (EGBP) are effective for rare diseases, the ideal diagnostic approach is debated. Limited research has explored reanalyzing raw ES and GS data post-negative EGBP results for diagnostics. Results: We analyzed complete ES/GS raw sequencing data from Mayo Clinic's Program for Rare and Undiagnosed Diseases (PRaUD) patients to assess whether supplementary findings could augment diagnostic yield. ES data from 80 patients (59 adults) and GS data from 20 patients (10 adults), averaging 43 years in age, were analyzed. Most patients had renal (*n*=44) and auto-inflammatory (*n*=29) phenotypes. Ninety-six cases had negative findings and in four cases additional genetic variants were found, including a variant related to a recently described disease (RRAGD-related hypomagnesemia), a variant missed due to discordant inheritance pattern (*COL4A3*), a variant with high allelic frequency (*NPHS2*) in the general population, and a variant associated with an initially untargeted phenotype (*HNF1A*). Conclusion: ES and GS show diagnostic yields comparable to EGBP for single-system diseases. However, EGBP's limitations in detecting new disease-associated genes underscore the necessity for periodic updates.

**Supplementary Information:**

The online version contains supplementary material available at 10.1186/s13023-024-03213-x.

## Background

Over the last decade, next-generation sequencing (NGS)-based tests have emerged as the first-line approach in diagnosing patients with rare diseases (RD). Clinical practice predominantly employs several NGS methodologies, including multi-gene panels (MGP) utilizing targeted gene enrichment, exome sequencing (ES) covering all known genes (approximately 1-2% of the genome), genome sequencing (GS) spanning a much broader genomic spectrum (50 to 100 times the content of ES, encompassing regulatory, intronic, and intergenic regions), and exome and genome-based targeted panels (EGBP) [1, 2]. MGP entails a focused analysis of a curated set of clinically significant genes, ensuring adequate coverage for the phenotype under consideration [3, 4]. While ES and GS offer comprehensive genomic analysis, they may necessitate supplementary measures to enhance coverage in regions with low mappability, as achieved in MGP through complementary methods like Sanger sequencing and qPCR, augmenting depth and coverage [5–8]. Despite potentially lower coverage compared to MGP, EGBP, which use in silico target selection, presents an adaptable alternative, characterized by its ability to swiftly modify gene content and expedite analysis, which is particularly advantageous in the evolving domain of genetics [4].

Clinical ES has demonstrated diagnostic rates ranging from 20% to 50%, showing a similar diagnostic yield aligning with the diagnostic efficacy of MGP approaches, contingent upon patient selection criteria [1, 9, 10]. Moreover, ES typically incurs higher costs compared to MGP and EGBP, potentially influencing provider preferences due to financial considerations [2]. The reanalysis of sequencing raw data stands out as a compelling strategy in instances where the initial diagnostic method yields negative or inconclusive results. Of note, approximately 30% of positive cases identified by GS following negative ES outcomes could have been detected through reevaluation of the ES raw data [11]. Furthermore, the integration of translational research, encompassing variant curation and research-driven initiatives, has shown promise in elevating diagnostic rates for cases with negative clinical ES results [12]. Nevertheless, there remains a scarcity of studies delineating the supplemental diagnostic value derived from reanalyzing ES and GS raw data in patients exhibiting negative findings in EGBP, particularly those presenting with a clearly defined clinical phenotype.

The Program for Rare and Undiagnosed Diseases (PRaUD) at Mayo Clinic provides comprehensive genomic-based clinical services for rare diseases (RD), seamlessly integrating genetic testing, research, and education into patient care across various specialized divisions and departments [13]. PRaUD adopts a first-tier diagnostic approach utilizing targeted MGP or customized EGBP. In this current study, we evaluated a cohort of 100 patients from PRaUD who received undiagnostic results from their custom EGBP. Our objective was to evaluate whether an in-depth analysis of the complete ES or GS raw sequencing data could uncover additional findings, potentially elevating the diagnostic yield for these patients.

## Materials and methods

### Patient cohort

This study used a convenience sampling method including patients evaluated by PRaUD-affiliated clinicians within five departments/divisions for whom ES/GS data were available. These individuals were suspected to exhibit a genetic cause for their observed phenotype, and their cases remained unresolved following the initial genetic assessment [13]. The assessments took place at Mayo Clinic campuses situated in Minnesota, Florida, and Arizona, spanning from December 2018 to August 2023. Patients demonstrating strong indicators of a genetic disorder—such as a positive family history, early onset of symptoms, heightened disease severity, and inconclusive results from EGBP testing—were directed towards ES or GS raw data analysis. The EGBP tests were conducted at CLIA-certified and CAP-accredited laboratories. For specific details regarding the gene content of each panel, please refer to Supplementary Table 1.

### Data management

All participants or legal guardians provided explicit written informed consent approved by the Mayo Clinic Institutional Review Board (IRB#19-003389). Protocols for data transfer and reprocessing were established in collaboration with the clinical laboratories to procure the available sequencing files (FASTQ, BAM, CRAM, and/or VCF). Information regarding sociodemographic attributes, clinical history, histopathological findings, and genetic analysis was extracted from electronic health records (EHRs) and securely stored in Redcap and scientific data management system (SDMS) HIPAA-compliant databases.

### Analysis of raw sequencing data

For the analysis of raw sequencing files, we utilized commercial genomic prioritization tools that operate through AI-driven graphical interfaces, requiring the input of VCF or BAM files, along with information on sex, age of onset, and Human Phenotype Ontology (HPO) terms. One of the softwares additionally provides automated reanalysis at specified intervals. Variants identified through automated reanalysis underwent manual scrutiny to determine their clinical relevance throughout the duration of the study. Variant curation included phenotypic congruence, in silico predictions, as well as insights from population cohort studies and literature search. Variants were categorized following the American College of Medical Genetics and Genomics/Association for Molecular Pathology guidelines, including updates until December 2022 [14]. Any pertinent genetic findings were subsequently deliberated with the PRaUD team for their clinical significance and for planning follow-up steps.

## Results

**Fig. 1:**
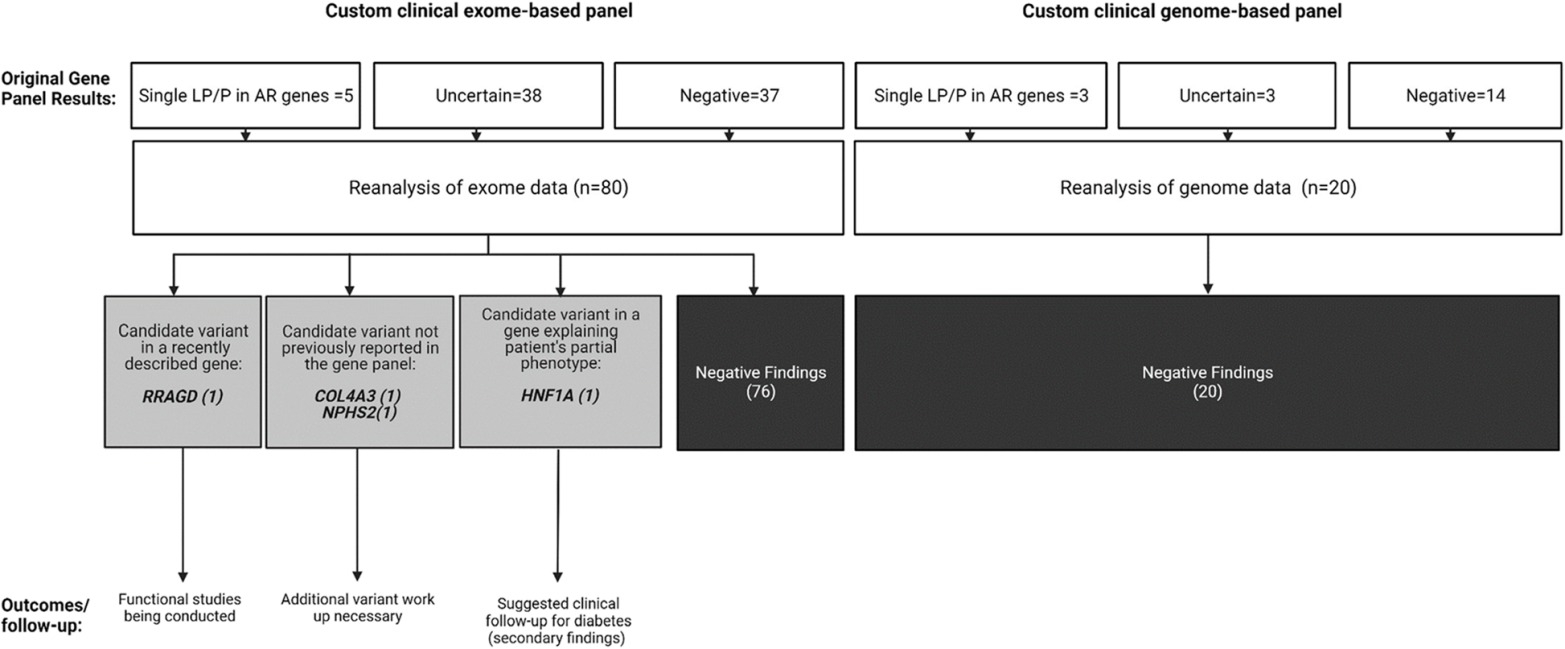
Results of the re-analyses of custom clinical exome and genome-based panels data of 100 patients with single-system diseases

ES data from 80 patients (59 adults, 47 females) and GS from 20 patients (10 adults, 13 males) were analyzed. The age of the patients at the time of genetic testing ranged from 4 to 81 years old, with a mean age of 43 years. The original EGBP reports for these patients yielded the following results: negative in 54 patients, containing a variant of uncertain significance (VUS) in genes of interest in 41 patients, and reported as positive (containing one likely pathogenic or pathogenic variant in a gene associated with an AR phenotype) in 8 patients as demonstrated in Figure 1**.** The median between the issuance of the clinical report and the subsequent reanalysis of the sequencing data was 12 months, with an interquartile range (IQR) spanning from 7 to 30 months. The majority of patients were referred from the Nephrology division (*n*=44), followed by Rheumatology (*n*=29), Endocrinology (n=13), and Pulmonary and Critical Care Medicine (*n*=12). The most common reasons for testing were auto-inflammatory syndrome (*n*=30) and focal segmental glomerulosclerosis (FSGS) (*n*=26). The complete reason for referral can be found in Table 1. Patients of African ancestry (three individuals) were evaluated for the *APOL1* (HGNC:618), G1 (NM_001136540:c.1024A>G, p.(Ser342Gly), and NM_001136540:c.1152T>G, p.(Ile384Met)) and G2 (NM_001136540:c.1160_1165delATAATT) polymorphic risk alleles due to the association with kidney disease within this population [15]. Demographic information can be found in Table 2 and Supplementary Table 2.

**Table 1:** Phenotypes of the individuals included in the study

**Phenotypes**	**Number of individuals (*n*=100)**
**Endocrinology**	
Short stature	6
MODY	6
Early Onset Osteoporosis	1
**Nephrology and Hypertension**	
Glomerulopathy	26
CAKUT	6
Kidney stones	5
Kidney cysts	4
Tubulointerstitial	2
Electrolyte imbalance	1
**Neurology**	
Ataxia	1
**Pulmonary**	
Interstitial lung disease	12
**Rheumatology and Infectious Diseases**	
Auto-inflammatory Syndromes	30

MODY: Maturity Onset Diabetes of the Young

CAKUT: Congenital Anomalies of the Kidney and Urinary Tract

**Table 2:** Demographic information

**Characteristic**	**Number of individuals (*n*=100)**
**Sex**	
Female	54
Male	46
**Race or ethnic group**	
White	92
African American/African	3
Other/Chose not to disclose	3
Asian	2
**Age at time of testing (years)**	
0-17	18
18-30	13
31-50	27
51-70	29
>70	13
**Age at onset of symptoms (years)**	
0-17	37
18-30	9
31-50	18
51-70	18
>70	3
Unknown	15
**Positive Family History**	
Yes	55
No	31
Not available	14
**NGS technology**	
ES	80
GS	20
**Time for re-analysis after the clinical report (months)**	
<12	47
12-24	23
24-36	13
>36	17

*ES* Exome sequencing, *GS* Genome sequencing

Upon re-analysis of the exome/genome data, no additional findings were identified in 96 individuals. In the remaining four (4%), additional findings were discovered. In one case, a variant in the *RRAGD* (HGNC:19903) gene was found, which is associated with a phenotype reported in the literature after the release of the original report. In two cases, variants that were part of the original EGBP were not reported by the clinical laboratory. This included a *COL4A3* (HGNC:2204) variant due to a discordant inheritance pattern and a variant in *NPHS2* (HGNC:13394), which was omitted due to its high population prevalence. In a fourth case, a likely pathogenic variant in *HNF1A* (HGNC:11621) was identified, which might explain the patient's partial phenotype. The summary of the key learning points of each case can be found in Table 3. Additionally, periodic automated re-analysis during the specified period flagged variants in 26 cases; however, after further review, these variants were deemed not relevant for the proband's phenotypes since they were primarily single VUS in recessive genes or in genes associated with multisystem syndromes that were flagged by the softwares because those syndromes encompass HPO terms included in the referral reason (data not shown).

**Table 3:** Summary of the key learning points of the cases with findings after analysis of genomic raw data

**Patient**	**Finding on raw data analysis**	**Learning points**
1	VUS in *COL4A3*	A careful review of the raw genomic data for unreported variants in genes of interest is essential as clinical laboratories follow different guidelines for variant interpretation and reporting.
2	VUS in *NPHS2*	
3	Variant in a GUS - *RRAGD*	The discovery of novel genes is a considerable challenge when utilizing a multi-gene panel approach. Regular update of the gene content is necessary.
4	LP variant in *HNF1A*	The multi-gene panel may not include genes associated with all phenotypes present in the proband. Selection of appropriate panel(s) or proper selection of the genes associated with all phenotypes is warranted.

*VUS* Variant of uncertain significance, *LP* Likely pathogenic, *GUS* Gene of uncertain significance

### Case vignettes

#### Case 1 – conflicting inheritance pattern

A 62-year-old Caucasian female patient presents with a medical history characterized by focal segmental glomerulosclerosis (FSGS) lesion in a renal biopsy at the age of 57. Family history reveals two paternal uncles with kidney disease, attributed to congestive heart failure and diabetes, respectively. Initial symptoms manifested around age 56, marked by edema, with an albumin level of 2.8 g/dL (Reference Range, RR: 3.2 - 4.6 g/dL) and creatinine of 0.8 mg/dL (RR: 0.59 - 1.04 mg/dL). At 57, a 24-hour urine collection showed 9 g of protein (RR: <229 mg/24 h) and albumin levels of 1.8 g/dL (RR: 3.5 - 5.0 g/dL). The biopsy confirmed segmental glomerulosclerosis, with negative immunofluorescence for various markers except focal segmental immunoreactivity with fibrinogen (2+). Electron microscopy revealed extensive effacement of visceral epithelial cell foot processes. Commencing treatment with an angiotensin-converting enzyme inhibitor and prednisone, later switched to cyclosporin, the patient faced additional challenges such as mild hyperlipidemia, with triglyceride levels at 160 mg/dL (RR: <150 mg/dL). Baseline creatinine fluctuated between 2.2 to 2.6 mg/dL (RR: 0.59 - 1.04 mg/dL) since age 60. Despite interventions, renal function decline prompted enrollment in a clinical trial with obinutuzumab. Investigation for genetic causes of FSGS lesion with an EGBP was initiated at age 58 and yielded a negative result. Subsequent re-analysis of the sequencing data detected the NM_000091.4: c.3182 G>A; p.(Gly1061Asp), variant of uncertain significance (VUS) in *COL4A3* (HGNC:2204)*,* a gene associated with recessive and dominant forms of Alport syndrome (MIM 203780 and 104200). This glycine substitution is identified in 49 alleles out of 248,632, with no homozygotes in gnomAD. Notably, similar substitutions (p.(Gly1023Arg), p.(Gly1035Val), p.(Gly1038Ser)) have been described as pathogenic or likely pathogenic in the same exon. The variant was clinically confirmed by the laboratory after initial oversight due to conflicting inheritance patterns, and family segregation studies were recommended for a comprehensive understanding of the variant's role in the proband's phenotype, especially if other family members exhibit biopsy-proven FSGS.

#### Case 2- variant prevalent in the general population

A 43-year-old male with renal failure and FSGS lesion on the kidney biopsy. The diagnosis of FSGS was established at the age of 26 prompted by the discovery of proteinuria during an insurance screening, including urinalysis. Analysis of a 24-hour urine collection at that time revealed a protein loss of 7.7 g/24 h (reference range <229 mg/24 h). Further laboratory investigations disclosed hypercholesterolemia (total fasting cholesterol 320 mg/dL, desirable <200 mg/dL), hypertriglyceridemia (fasting triglycerides 576 mg/dL, reference range <150 mg/dL), and plasma albumin levels of 2.9 g/dL (reference range 3.4 to 5.4 g/dL). There was no familial history of similar symptoms. At the age of 38, an EGBP identified a pathogenic variant in exon 8 of *NPHS2* (NM_014625.3: c.948delT; p. (Ala317LeufsTer31)) associated with autosomal recessive nephrotic syndrome type 2 (MIM 600995). This variant was deemed pathogenic by multiple clinical laboratories (ClinVar ID: 188990). The initial report did not mention a second hit in this gene, and the exome sequencing data showed no evidence of multi-exon deletion/duplication involving this gene. During quality control background testing for the EGBP, the clinical laboratory identified a likely duplication of the X chromosome, consistent with Klinefelter syndrome, a finding confirmed by karyotype analysis. This secondary discovery was considered causative for the patient's history of azoospermia and tall stature. Subsequent re-analysis of raw ES data uncovered a second variant in *NPHS2* (HGNC:13394; NM_014625.4:c.686G>A; p.(Arg229Gln)), not previously reported by the clinical laboratory. Despite its prevalence in the general population (8,538 alleles out of 282,294 in gnomAD, including 186 homozygotes) and uncertain in silico predictions (REVEL = 0.58), this variant has been traditionally documented in the literature as disease-causing, depending on the variant observed on the other chromosome [16]. At the age of 42, the patient underwent a successful renal transplant from a living donor, experiencing an uneventful postoperative course with immediate kidney allograft function.

#### Case 3 – novel gene-disease association

A 46-year-old female of Ashkenazi Jewish descent with nephrolithiasis, hypomagnesemia, and hypokalemia. She has family history of the maternal grandmother experiencing nephrolithiasis, and her mother and two maternal uncles exhibiting electrolyte imbalances suggestive of Gitelman syndrome. Born via C-section at full term, her delivery was complicated by her mother's hypokalemia-induced cardiac arrest. At 8-10 months, she developed a urinary tract infection, with nephrolithiasis diagnosed at 15 months, necessitating a partial nephrectomy for stone removal. Throughout childhood, she frequently experienced urinary tract infections, responding well to sulfamethoxazole and trimethoprim therapy. In adulthood, recurrent severe pyelonephritis episodes ensued. Paresthesias developed, accompanied by intermittent hypokalemia, hypomagnesemia, and occasionally hypocalcemia. Treatment with magnesium and potassium replacement therapy was initiated. At 40, pancreatitis episodes exacerbated by pyelonephritis and sepsis led to a diabetes diagnosis, prompting a switch from metformin to insulin. A kidney ultrasound at 42 revealed medullary nephrocalcinosis with bilateral renal calculi, non-obstructive. Genetic testing at 43, conducted in November 2019 via EGBP, yielded negative results. However, a reanalysis of the ES data in January 2022 identified a VUS in *RRAGD* (Ras-related GTP binding D, HGNC:19903), a gene newly associated in November 2021 with hypomagnesemia, tubulopathy, and dilated cardiomyopathy. Variants in this gene have been described as causing electrolyte-losing tubulopathy and dilated cardiomyopathy due to the activation of mTOR signaling, suggesting a crucial role for Rag GTPase D in renal electrolyte regulation and cardiac function [17]. Patient’s variant is absent in population databases and has a high REVEL score of 0.67, predicting it to be deleterious. Despite normal echocardiogram results, further functional testing and family segregation studies are underway in collaboration with the original report authors due to the unique findings in this case [17].

#### Case 4 – secondary phenotype

A 44-year-old female with symptoms of hypokalemia and polyuria, with a notable family history of diabetes in her mother. The hypokalemia was initially identified at the age of 36 during an angina pectoris evaluation, prompted by an ECG revealing a prolonged QT interval. At that time, her potassium levels measured 2 mmol/L (reference range 3.6 - 5.2 mmol/L). Concurrently, she was diagnosed with polyuria, experiencing urine output ranging from 5 to 10 liters daily. Hypokalemic manifestations included intermittent neurological symptoms, reduced mentation, impaired concentration, dizziness, and headaches. Alongside hypokalemia, she presented with hypomagnesemia and chronic constipation, necessitating a rotating laxative regimen, possibly linked to her electrolyte imbalance. Her diabetes workup in 2021 displayed abnormal hemoglobin A1C levels at 5.9% (reference range <=5.6%) and estimated average glucose levels of 191 mg/dL (70 - 180 mg/dL). Following initiation of semaglutide treatment, these levels normalized during her latest assessment. EGBP at age 41, prompted by her history of hypokalemia and polyuria, initially yielded negative results. However, re-analysis of exome raw data revealed a likely pathogenic variant in *HNF1A* (NM_000545.8) c.1745A>G, p.(His582Arg) not previously assessed in the nephrology-focused EGBP. This variant is present in 13 alleles out of 240,596 in gnomAD and it is predicted deleterious (REVEL=0.69). It has been previously identified with suboptimal function in in vitro assays, and classified as a strong type 2 diabetes risk modifier in maturity-onset diabetes of the young (MODY) studies [18, 19]. This information was conveyed to the clinical team for further exploration of her diabetes diagnosis and assessment of the variant's significance in her family's diabetes history through segregation studies.

## Discussion

Determining the first-tier genetic testing approach requires consideration of the cost-effectiveness of the ordered NGS technique. This becomes particularly important in subspecialty clinics where insurance companies and other payers often seek clarification [20]. Furthermore, comprehensive genomic approaches may require additional efforts regarding result interpretation and education of healthcare providers, patients, and their families about the findings, particularly related to the number of VUS in unrelated genes.

In a study focusing on Nephrology patients who underwent MGP testing, the initial diagnostic yield was 20%, which increased to 30% after ES, with additional findings in kidney disease-related genes not included in the panel and identification of *APOL1* risk alleles not reported due to high population frequency [21]. In our cohort, one 77-year-old African American individual is homozygous for the G1 risk allele. Although a kidney biopsy was not done to rule out FSGS lesion, the patient was referred for kidney cysts which is not a common *APOL1*-related finding. Of note, most of the cases with new findings in that study were initially evaluated for atypical hemolytic uremic syndrome (*n*=224) which might be caused by complement and non-complement genes and present with ESRD. Considering other kidney phenotypes such glomerulopathies, ES yielded additional findings in 5 out of 69 patients (7%) [21]. When examining the diagnostic yield for PRaUD’s EGPB in nephrology cases, a diagnosis was achieved for 50 families (30.7%). Notably, there was a higher yield for tubulointerstitial kidney disease (53.3%, 8 of 15) and glomerulopathies (31%, 31 of 100) [22]. It is worth mentioning that the variance between studies can be attributed to patient selection criteria, the involvement of a multidisciplinary team facilitating discussions on the follow-up of VUS, and research opportunities, for example.

Cases 1 and 2 serve as examples of variants found in genes initially present in the phenotype-specific EGBP but were not initially reported by the clinical laboratory. Pathogenic variants in *COL4A3* (HGNC:2204) are recognized to be associated with *COL4A*-related diseases, commonly referred to as Alport syndrome. This genetic condition can be inherited in an autosomal dominant manner, often manifesting with milder symptoms compared to the autosomal recessive form [23, 24]. Family segregation studies and further clinical screening for *COL4A3*-related extra-renal symptoms, such as deafness were recommended. This information could also prove valuable in future transplant decisions, as testing potential donors for the presence of this variant may be advisable [25]. Similar attention is warranted for *NPHS2* (HGNC:13394), considering its association with nephrotic syndrome [26]. The NM_014625.4:c.686G>A; p.(Arg229Gln) variant, initially omitted from the clinical EGBP report, is noteworthy in the literature due to its pathogenicity being dependent on the presence of a trans-associated pathogenic variant in exon 7 or exon 8. It primarily causes disease when paired with a variant that exerts a dominant negative effect, and it does not cause the disease when in homozygous state [16]. The effect of this variant in conjunction with the previously reported pathogenic variant for Case 2 - p.(Ala317LeufsTer31) remains unclear, although the frameshift variant is located where other causative variants have been reported. This truncating variant is predicted to disrupt the oligomerization of podocin, encoded by *NPHS2* (HGNC:13394), which does not align with a complementary pathogenic effect for p.(Arg229Gln) [16]. While the patient's phenotype remains uncertain at this moment, knowledge of the presence of this variant holds significance for genetic counseling and offers opportunities for further re-analysis in the light of additional case reports or functional evidence [27].

Case 3 serves as an illustrative example of a patient who received a diagnosis after manual re-analysis, uncovering a variant in a newly described gene *(RRAGD,* HGNC:19903) that had not been previously screened through the phenotype-specific EGBP. The identification of new genes associated with diseases poses a significant challenge when employing a MGP approach, given the rapid evolution of knowledge regarding gene-disease associations. A study involving pediatric patients from non-genetic subspecialty clinics demonstrated GS diagnostic rate of 41%, encompassing several emerging disease genes not previously identified by other genetic tests [28]. This highlights the need for ongoing monitoring and revision of content in MGP and EGBP, especially when new disease genes are identified. Such decisions may necessitate consultations with disease experts and regular literature surveys [28]. The importance of re-analysis extends beyond unsolved cases but also for cases previously considered resolved but with insufficient evidence. For instance, in a study of a follow-up cohort comprising 152 consanguineous families with developmental disorders, re-analysis of ES data after 5 years revealed 5 new gene-disease associations and led to the reclassification of 10 variants previously reported as pathogenic [29].

Case 4 serves as an example of how the selection of a MGP may not always include genes associated with all of the proband's phenotypes, potentially resulting in an incomplete representation of the full differential diagnosis for the case [1, 28]. Despite the primary phenotype being an electrolyte imbalance, the identification of a variant in a MODY-associated gene is clinically significant as it might explain the hyperglycemia, polyuria, and positive family history of diabetes. A recent study comparing genetic diagnostic approaches to MODY sheds light on this scenario. The study, involving 146 patients diagnosed with obesity or diabetes who underwent both MGP and ES, revealed similar diagnostic yield for this phenotype between the two techniques, amounting to 34.9%, with ES reporting additional variants in two novel genes [30]. Case 4 highlights the importance of considering different diagnoses for the same phenotype, as such an approach may enable the inclusion of all potential candidate genes in the investigation as previous cohorts described the diagnosis of more than one independent monogenic condition in approximately 3%-7% of the cases [12, 31, 32]. This has the potential to enhance the diagnostic yield of first-tier genetic investigations, a critical consideration when patients have limited opportunities for subsequent genetic tests [33].

Automated re-analysis emerges as crucial approach requiring less effort, offering an advantage for periodic systematic re-annotation of genome-wide variants [34]. In this study, one of the tools employed for manual re-analysis offered automated periodic re-analysis of the raw data but did not yield significant findings. Instead, after additional manual review, it mainly flagged VUS in genes associated with multi-system syndromes that contains phenotypes related to the reason for referral. For example, a VUS in *HERC2* was flagged due to its potential association with unexplained fevers within the broad clinical spectrum of Intellectual Developmental Disorder, Autosomal Recessive 38 syndrome, despite the absence of any other symptoms in the patient. Given that our cohort primarily consisted of adults with single-system involvement, our outcomes differ from those of a study that employed automated re-analysis for GS cases. The latter revealed positive findings in 31% (5 out of 16) of undiagnosed pediatric cases, with two of them linked to variants found in genes initially omitted from the original panel due to incomplete initial phenotyping [35].

The analysis of ES/GS data of 100 unsolved cases with single-system diseases, following EGPB revealed additional findings in four cases (4%), with two of them involving genes already included in the clinical panel, one in a novel gene primarily associated with the reason for referral and one in a gene not included in the panel because it was related to a secondary phenotype. One reason for the limited increase in the solve rate after reviewing ES/GS data could be related to the prevalence of auto-inflammatory syndromes in our cohort which is known to have a low diagnostic yield, attributed to unspecific phenotypes [36]. Moreover, our cohort includes several Nephrology cases. Kidney genetic diseases have more specific phenotypes and more clear gene-disease associations than other diseases included in this study so the clinical MGP were comprehensive and included most of the known genes expressed in the kidney [20]. Conversely, we included fewer cases from the Neurology department which usually encompass phenotypes known to have higher solve rate after ES/GS [35, 37]. The study highlighted the importance of targeted, phenotype-specific EGBP to maintain clinical sensitivity while minimizing the burden of analyzing a larger number of variants in genes that might not be related to the main phenotype. Noteworthy, cases 1 and 2 underscore the importance of a careful review of the data for unreported variants in genes of interest since clinical laboratories might follow different guidelines for variant reporting. Furthermore, identifying new disease-associated genes poses a significant challenge when employing an EGPB approach, given the evolving knowledge of gene-disease associations.

## Conclusion

Our experience highlights that employing an EGBP tailored to a specific phenotype, administered by a multidisciplinary team of experts, can yield diagnostic results comparable to those obtained through ES and GS sequencing. Notably, our study indicated that clinical laboratories rarely missed diagnoses, and the potential limitation of EGBP was the discovery of new gene-disease associations and genes for differential diagnosis. These findings underscore the importance of a targeted approach in patients with single-system diseases, supporting the notion that EGBP serves as a valuable and cost-effective alternative to broader and more expensive NGS techniques.

### Supplementary Information

Supplementary Material 1.

Supplementary Material 2.

## Data Availability

The datasets generated and/or analyzed during the current study are not publicly available but are available from the corresponding author on reasonable request.

## Abbreviations

EGPB: Exome and genome-based targeted panel
ES: Exome Sequencing
FSGS: Focal segmental glomerulosclerosis
MGP: Multi-Gene Panel
GS: Genome sequencing
NGS: Next Generation Sequencing
NS: Nephrotic syndrome
PRaUD: Program for Rare and Undiagnosed Diseases
VUS: Variant of uncertain significance
