# Supplementary material for: Diagnostic yield of exome and genome sequencing after non-diagnostic multi-gene panels in patients with single-system diseases
Source: Orphanet J Rare Dis. 2024 May 24;19:216. doi: 10.1186/s13023-024-03213-x (PMC11127317; doi:10.1186/s13023-024-03213-x)
Supplement: Supplementary file 1 — Supplementary Material 1. [file 13023_2024_3213_MOESM1_ESM.docx]

| Gene Sets per Division/Department |
| --- |
| **Endocrinology – Short Stature (53 genes)** |
| *ACAN, ADAMTS10, BRAF, BTK, CBL, CCDC8, CDT1, COMP, CUL7, FGF8, FGFR1, FGFR3, GH1, GHR, GHRH, GHRHR, GHSR, GLI2, GNAS, HESX1,IGF1, IGF1R, IGF2, IGFALS, IHH, IKBKB, KRAS, LHX3, LHX4, LZTR1, NPPC, NPR2, NRAS, OBSL1, OTX2, PAPPA2, PCNT, PITX2, POU1F1, PROP1, PTH1R, PTHLH, PTPN11, RAF1, RASA2, RIT1, RRAS, SHOC2, SHOX, SOS1, SOX2, SOX3, STAT5B.*  **Endocrinology – Osteoporosis (148 genes)** *ACVR1, AIRE, ALPL, AMER1, ANKH, AP2S1, ASCC1, B3GAT3, B4GALT7, BAZ1B, BMP1, CA2, CASR, CDC73, CDK5RAP2, CDKN1A, CDKN1B, CDKN2B, CDKN2C, CLCN5, CLCN7, COL1A1, COL1A2, COMP, CREB3L1, CRTAP, CTSK, CYP24A1, CYP27B1, CYP2R1, CYP3A4, DMP1, ELN, ENPP1, EXT1, EXT2, FAH, FAM20C, FAM46A, FBN1, FERMT3, FGF23, FGFR1, FGFR2, FGFR3, FKBP10, FLNB, GALNT3, GATA3, GBA, GCM2, GNA11, GNAS, GPAA1, HAMP, HFE, HNRNPC, HSPG2, IFITM5, IKBKG, KL, LEMD3, LRP4, LRP5, LRP6, MBTPS2, MEN1, MEPE, NBAS, NTRK1, OSTM1, P4HB, PAPSS2, PHEX, PLEKHM1, PLOD2, PLS3, POR, PPIB, PRKAR1A, PTH, PTH1R, PTPN11, RET, RUNX2, SAMD9, SEC24D, SERPINF1, SERPINH1, SFRP4, SGMS2, SH3PXD2B, SHOX, SLC26A2, SLC29A3, SLC2A2, SLC34A1, SLC34A2, SLC34A3, SLC40A1, SLC7A7, SLC9A3R1, SNX10, SOST, SOX3, SOX9, SP7, SPARC, STX16, TAPT1, TBCE, TBX1, TCIRG1, TFR2, TGFB1, TMEM38B, TNFRSF11A, TNFRSF11B, TNFSF11, TRIP11, TRIP4, TRPM6, TRPV6, VDR, WNT1, XYLT2, HJV [HFE2], MESD [MESDC2], P3H1 [LEPRE1].*  **Endocrinology – Maturity onset diabetes of the young (MODY) (85 genes)** *ABCC8, ACAT1, ACSF3, AGL, AIRE, AKT2, AKT3, ALDOA, ALDOB, APPL1, BLK, CDKN1C, CEL, CISD2, CP, DNAJC3, DUT, EIF2AK3, ENO3, EPM2A, FBP1, FOXP3, G6PC1, GAA, GATA4, GATA6, GBE1, GCK, GLIS3, GLUD1, GYG1, GYS1, GYS2, HADH, HK1, HMGCL, HMGCS2,HNF1A, HNF1B, HNF4A, IER3IP1, IL2RA, INS, INSR, KCNJ11, KDM6A, KLF11, KMT2D, LAMP2, LDHA, LRBA, MNX1, MPV17, NEUROD1, NEUROG3, NHLRC1, NKX2-2, OXCT1, PAX4, PC, PCBD1, PCK1, PDX1, PFKM, PGAM2, PGK1, PGM1, PHKA1, PHKA2, PHKB, PHKG2, PLAGL1, PRKAG2, PRKAG3, PTF1A, PYGL, PYGM, RBCK1, RFX6, SH2B1, SLC16A1, SLC19A2, SLC2A2, SLC37A4, STAT3, TRMT10A, UCP2, WFS1, ZBTB20, ZFP57.* |
| **Nephrology (366 genes)** |
| *ACE, ACTN4, ADAMTS13, ADCY10, AGT, AGTR1, AGXT, AHI1, ALG1, ALG8, ALMS1, ALPL, ANKS6, ANLN, ANOS1, APOA1, APOE, APOL1, APRT, AQP2, ARHGAP24, ARHGDIA, ARL13B, ARL6, ATP6V0A4, ATP6V1B1, ATP7B, ATXN10, AVP, AVPR2, B2M, B9D1, B9D2, BBIP1, BBS1, BBS10, BBS12, BBS2, BBS4, BBS5, BBS7, BBS9, BICC1, BMP4, BMP7, BSND, C2CD3, C3, C8orf37, CA2, CACNA1D, CACNA1H, CASR, CC2D2A,CD151, CD2AP, CD46, CDC5L, CEP104, CEP120, CEP164, CEP290, CEP41, CEP83, CFB, CFD, CFH, CFHR1, CFHR3, CFHR4, CFHR5, CFI, CHD1L, CHD7, CHRM3,CLCN5, CLCNKA, CLCNKB, CLDN14, CLDN16, CLDN19, CNNM2, COL4A1, COL4A3, COL4A4, COL4A5, COL4A6, COQ2, COQ6, CRB2, CREBBP, CSPP1, CTNS, CUBN, CUL3 ,CYP11B1, CYP11B2, CYP24A1, DACH1, DCDC2, DDX59, DGKE, DGKH, DHCR7, DHTKD1, DLC1, DLG1, DMP1, DNAJB11, DSTYK, DYNC2H1, DZIP1L, E2F3, EGF, EHHADH, EMP2, ENPP1, EVC, EVC2, EYA1, F2, FAH, FAM20A, FAN1, FAT1, FGA ,FGF20, FGF23, FGFR1, FGFR2, FN1, FOXI1, FOXP1, FRAS1, FREM1, FREM2, FXYD2, GANAB, GATA3, GDNF, GFRA1, GLA, GLI3, GLIS2, GPC3, GREM1, GRHPR ,GRIP1, GSN, HNF1B, HNF4A, HOGA1, HPRT1, HPSE2, HSD11B2, IFT122, IFT140, IFT172, IFT27, IFT43, IFT52, IFT57, IFT74, IFT80, IFT81, IGFBP7, INF2, INPP5E, INVS, IQCB1, ITGA3, ITGA8, ITGB4, JAG1, KANK1, KANK2, KANK4, KCNJ1, KCNJ10 ,KCNJ5, KIAA0556, KIAA0586, KIAA0753, KIF12, KIF14, KIF7, KL, KLHL3, LAMA5, LAMB2, LMNA, LMX1B, LRIG2, LRP5, LYZ, LZTFL1, MAGI2, MEFV, MKKS, MKS1, MYH9, MYO1E, NEDD4L, NEK1, NEK8, NLRP3, NOTCH2, NPHP1, NPHP3, NPHP4, NPHS1, NPHS2, NR3C2, NUP107, NUP133, NUP160, NUP205, NUP85, NUP93, NXF5, OCRL, OFD1, OXGR1, PAX2, PAX8, PBX1, PDE6D, PDSS2, PHEX, PKD1, PKD2, PKHD1, PLCE1, PLCG2, PLG, PMM2, POC1A, POC1B, PODXL, POMT2, PRKCSH, PRPS1 ,PTPRO, REN, RET, ROBO2, RPGRIP1L, SALL1, SALL4, SARS2, SCARB2, SCLT1 ,SCN10A, SCNN1A, SCNN1B, SCNN1G, SDCCAG8, SEC61A1, SEC61B, SEC63, SEMA3E, SGPL1, SIX1, SIX2, SIX5, SLC12A1, SLC12A3, SLC13A2, SLC17A5, SLC22A12, SLC26A1, SLC2A2, SLC2A9, SLC34A1, SLC34A3, SLC3A1, SLC41A1, SLC4A1, SLC4A4, SLC5A1, SLC5A2 ,SLC6A19, SLC7A13, SLC7A7, SLC7A9, SLC9A3R1, SLIT2, SMARCAL1, SOX17, SOX9, SPP1, SRGAP1, TBX18, TCTN1, TCTN2, TCTN3, THBD, TMEM107, TMEM138 ,TMEM216 ,TMEM231, TMEM237, TMEM67, TNFRSF1A, TNXB, TRAF3IP1, TRAP1, TRIM32, TRPC6, TRPM6, TRPV5, TSC1, TSC2, TTC21B, TTC8, TTR, UMOD ,UPK3A ,VDR ,VHL ,VIPAS39 ,VPS33B ,WDPCP ,WDR19 ,WDR34 ,WDR35, WDR60, WDR72 ,WDR73, WNK1, WNK4, WNT4, WT1, XDH, XPNPEP3, XPO5, ZMPSTE24, ZNF423, COQ8B [ADCK4], CPLANE1 [C5orf42].*  **Neurology (296 genes)**  *AAAS, AARS1, ABCB7, ABHD12, ACO2, COQ8A, ADPRS, AFG3L2, AGTPBP1, AHI1, ALAS2, ALDH5A1, ALS2, AMPD2, ANGPTL3, ANO10, AP1S2, APOB, APTX, ARL13B, ARL6, ARSA, ASAH1, ATCAY, ATL1, ATM, ATN1, ATP1A3, ATP2B3, ATP8A2, ATXN1, ATXN10, ATXN2, ATXN3, ATXN7, ATXN8OS, B9D1, BBS1, BBS10, BBS12, BBS2, BBS4, BBS5, BBS7, BBS9, BEAN1, BSCL2, TWNK, C12orf4, C12orf65, C19orf12, CPLANE1, CA8, CACNA1A, CACNA1G, CACNA2D2, CACNB4, CAMTA1, CAPN1, CASK, CC2D2A, CCDC88C, CEP290, CEP41, CHMP1A, CHP1, CHORDC1, CLCN2, CLN3, CLN5, CLN6, CLP1, CLPP, COA7, COASY, COG5, COQ2, COQ4, COQ6, COQ9, COX20, CP, CSTB, CWF19L1, CYP27A1, CYP2U1, CYP7B1, DAB1, DARS2, DDHD2, DHPS, DMXL2, DNAJC19, DNAJC5, DNMT1, DOCK3, DYNC1H1, EBF3, EEF2, EIF2B1, EIF2B2, EIF2B3, EIF2B4, EIF2B5, ELOVL4, ELOVL5, EPM2A, EXOSC3, FA2H, FBXL4, FDXR, FGF14, FLVCR1, FMR1, FOLR1, FXN, GALC, GBA2, GFAP, GJC2, GOSR2, GPAA1, GRID2, GRM1, GSS, PRNP, HARS2, DTD1, HEPACAM, HEXA, HEXB, HIBCH, HTT, SLC6A4, INPP5E, PMPCA, IRF2BPL, ITM2B, ITPR1, KCNA1, KCNC3, KCND3, KCNJ10, KIF1A, KIF1C, KIF5A, KIF7, LAMA1, LARS2, LMNB1, LRPPRC, MARS2, MECR, MKKS, MKS1, MLC1, MYL1, MMACHC, MME, MRE11, MSTO1, MT-ATP6, MTFMT, MTPAP, MTTP, MT-TP, MVK, NAGLU, NDUFAF6, NDUFS1, NDUFS2, NDUFS4, NDUFS7, NDUFS8, NDUFV1, NHLRC1, NKX6-2, NOL3, NOP56, NOTCH3, NPC1, NPC2, NPHP1, NUBPL, OFD1, OPA1, MED12, OPA3, OPHN1, PAX2, PAX6, PCLO, PDSS1, PDSS2, PDYN, PEX10, PEX16, PEX7, PHYH, PIK3R5, PLA2G6, PLP1, PMM2, PNKD, PNKP, PNPLA6, POLG, POLR3A, POLR3B, POLR3K, PPP2R2B, PRICKLE1, PRKCG, PRRT2, PUM1, RARS2, RELN, RFC1, SLC19A1, RNF170, RNF216, RORA, RPGRIP1L, RRM2B, RUBCN, SACS, SAR1B, SCN8A, SCYL1, CCL13, SEPSECS, SERAC1, SETX, SIL1, SLC16A2, SLC1A3, SLC20A2, SLC25A46, SLC2A1, SLC52A2, SLC52A3, SLC9A1, SLC9A6, SNX14, SPAST, SPG11, SPG7, SPR, TACR1, SPTBN2, SRD5A3, STUB1, SYNE1, SYT14, TBP, TCTN1, TCTN2, TCTN3, TDP1, TGM6, TMEM138, TMEM216, TMEM231, TMEM237, TMEM240, TMEM67, TPP1, ACD, TRIM32, TSEN2, TSEN34, TSEN54, TTBK2, TTC19, TTC8, TTPA, TUBB4A, TYMP, UBA5, UBR4, UBTF, UCHL1, VAMP1, VLDLR, VPS13D, VPS53, VRK1, WASHC5, WDPCP, WDR73, WDR81, WFS1, WWOX, XRCC1, ZFYVE26, ZNF423* |
| **Pulmonary and Critical Care Medicine (35 genes)** |
| *ABCA3, ACD, CSF2RA, CSF2RB, CTC1, DKC1, ELMOD2, FLCN, GBA, HPS1, HPS4, NAF1, NF1, NHP2, NOP10, PARN, RTEL1, SFTPA1, SFTPA2, SFTPB, SFTPC, SLC34A2, SLC7A7, SMPD1, STAT3, TERC, TERT, TINF2, TLR3, TMEM173, TOLLIP, TSC1, TSC2, USB1, WRAP53* |
| **Rheumatology (116 genes)** |
| *ACP5, ADA, ADA2[CECR1], ADAM17, ADAR, AICDA, AP3B1, AP3D1, ARPC1B, ASAH1, BTK, C1QA, C1QB, C1QC, CARD11, CARD14, CASP10, CD27, CD3G,, CD40LG, CD55, CD70, COPA, CTLA4, CTPS1, CYBA, CYBB, DCLRE1C, DDX58, DKC1, DNASE1, DNASE1L3, DNASE2, DOCK8, EGFR, ELANE, FADD, FOXP3, G6PC3, GATA2, GLA, ICOS, IFIH1, IL10, IL10RA, IL10RB, IL1RN, IL21, IL2RA, IL2RG, IL36RN, ISG15, ITCH, ITGB2, ITK, LACC1, LIG4, LPIN2, LRBA, LYN, LYST, MAGT1, MEFV, MVK, NCF2, NCF4, NCSTN, NFAT5, NLRC4, NLRP1, NLRP12, NLRP3, NOD2, OTULIN, PIK3CD, PIK3R1, PLCG2 ,POMP ,PRF1 ,PRKCD ,PSENEN ,PSMA3 ,PSMB3 ,PSMB4 ,PSMB8, PSMB9, PSTPIP1, RAB27A, RAG1, RAG2, RBCK1, RNASEH2A, RNASEH2B, RNASEH2C, RTEL1, SAMHD1, SH2D1A ,SH3BP2, SKIV2L, SLC29A3, SLC37A4, STAT1, STAT3, STIM1, STX11, STXBP2, TMEM173, TNFAIP3, TNFRSF1A, TREX1, TRNT1, TTC7A, UNC13D, WAS, XIAP, ZAP70 .* |

**Supplementary Table 1**: Genes present in the panels per division
