# Supplementary material for: Diagnostic yield of exome and genome sequencing after non-diagnostic multi-gene panels in patients with single-system diseases
Source: Orphanet J Rare Dis. 2024 May 24;19:216. doi: 10.1186/s13023-024-03213-x (PMC11127317; doi:10.1186/s13023-024-03213-x)
Supplement: Supplementary file 2 — Supplementary Material 2. [file 13023_2024_3213_MOESM2_ESM.pdf]

Supplementary Table 2: Demographic information

Patient Number	Sex	Current age (years)	Race	Division_1	Categories	Age at onset of symptoms	Positive family history?	EGBP report	Finding in the EGBP report (Gene/ Inheritance/ c./p./transcript/zygosity)	Difference between report and re-analysis (months)	Additional findings
1	Female	19	White	Endocrinology	Short Stature	Childhood	Unknown	Negative Findings		7	
2	Male	8	White	Endocrinology	Short Stature	Childhood	Unknown	Uncertain Clinical Significance	*LHX4*/Autosomal Dominant/c.65 T>C/p.V22A/NM_033343.3/1: 180199729/Heterozygous/Father/Variant of uncertain significance	8	
3	Female	16	White	Endocrinology	Short Stature	Childhood	Unknown	Negative Findings		7	
4	Male	5	White	Endocrinology	Maturity Onset Diabetes of the Young (MODY)	Childhood	No	Negative Findings		7	
5	Male	63	White	Neurology	Ataxia	Adulthood (51-70)	Unknown	Negative Findings		12	
6	Male	37	White	Endocrinology	Early Onset Osteoporosis	No	Yes	Uncertain Clinical Significance	*CYP24A1*/Autosomal Recessive/c.73 C>G/p.P25A/NM_000782.4/20:52790046/Heterozygous/Unknown/Variant of uncertain significance	1	
7	Female	17	White	Endocrinology	Short Stature	Childhood	Yes	Uncertain Clinical Significance	*OBSL1*/Autosomal Recessive/c.3947A>G/p.(Q1316R)/NM_015311.2/Heterozygous/Unknown/Variant of uncertain significance	3	
8	Female	10	White	Endocrinology	Short Stature	Neonatal	Yes	Negative Findings		2	
9	Male	14	White	Endocrinology	Short Stature	Childhood	No	Negative Findings		15	
10	Male	70	White	Endocrinology	Maturity Onset Diabetes of the Young (MODY)	Adulthood (18-30)	Yes	Negative Findings		3	
11	Male	36	White	Endocrinology	Maturity Onset Diabetes of the Young (MODY)	Adulthood (31-50)	Yes	Negative Findings		10	
12	Male	16	White	Endocrinology	Maturity Onset Diabetes of the Young (MODY)	Childhood	Yes	Negative Findings		3	
13	Male	33	White	Endocrinology	Maturity Onset Diabetes of the Young (MODY)	Adulthood (18-30)	Yes	Positive Findings	*WFS1*/Autosomal Recessive/c.1558C>T/p.Gln520Ter/NM_006005.3/Heterozygous/Unknown/Pathogenic	5	
14	Male	11	White	Endocrinology	Maturity Onset Diabetes of the Young (MODY)	Childhood	Yes	Negative Findings		27	
15	Female	76	Black or African American	Nephrology and Hypertension	Electrolyte imbalance	Unknown	Yes	Negative Findings		19	
16	Male	19	White	Infectious Diseases	Auto-inflammatory Syndromes	Childhood	No	Positive Findings	*UNC13D*/Autosomal Recessive/c.2298+1G>A/IVS23+G>A/NM_199242.2/17: 73830405/Heterozygous/Unknown/Likely pathogenic;CD27/Autosomal Recessive/c.778 C>T/p.P260S/NM_001242.4/12: 6560553/Heterozygous/Unknown/Variant of uncertain significance; *LYST*/Autosomal Recessive/c.6238 G>C/p.A2080P/NM_000081.2/1: 235926035/Heterozygous/Unknown/Variant of uncertain significance;*MAGT1*/X-linked/c.1002 G>A/p.M334I/NM_032121.5/X: 77086388/Hemizygous/Unknown/Variant of uncertain significance;*SLC7A7*/Autosomal Recessive/c.999 G>A/p.R333=/NM_001126106.2/14: 23244749/Heterozygous/Unknown/Variant of uncertain significance	40	
17	Female	59	White	Nephrology and Hypertension	Glomerulopathy	Adulthood (18-30)	Unknown	Uncertain Clinical Significance	*CASR*/Autosomal Dominant/Recessive/c.134 C>A/p.A45E/NM_000388.3/3: 121973170/Heterozygous/Unknown/Variant of uncertain significance	29	
18	Female	65	White	Nephrology and Hypertension	Glomerulopathy	Adulthood (31-50)	Yes	Uncertain Clinical Significance	*LAMA5*/Autosomal Dominant/Recessive/c.4105 C>T/p.R1369W/NM_005560.3/20: 60904847/Heterozygous/Unknown/Variant of uncertain significance; *LAMA5*/Autosomal Dominant/Recessive/c.896 G>T/p.R299L/NM_005560.3/20: 60926820/Heterozygous/Unknown/Variant of uncertain significance;*NPHP4*/Autosomal Dominant/Recessive/c.271 T>C/p.F91L/NM_015102.3/1: 6038338/Heterozygous/Unknown/Variant of uncertain significance	41	
19	Female	65	White	Nephrology and Hypertension	Glomerulopathy	Adulthood (51-70)	No	Negative Findings		49	
20	Male	40	White	Nephrology and Hypertension	Glomerulopathy	Unknown	Yes	Negative Findings		33	
21	Female	63	White	Nephrology and Hypertension	Glomerulopathy	Adulthood (51-70)	Yes	Negative Findings		30	COL4A3 (NM_000091.4) c.3182 G>A; p.Gly1061Asp
22	Female	59	White	Nephrology and Hypertension	Cysts	Adulthood (31-50)	Yes	Negative Findings		33	

Supplementary Table 2: Demographic information

23	Male	51	White	Nephrology and Hypertension	Glomerulopathy	Adulthood (31-50)	Yes	Negative Findings		36	
24	Female	46	White	Nephrology and Hypertension	Glomerulopathy	Childhood	Yes	Uncertain Clinical Significance	*NPHP4*/Autosomal Dominant/Recessive/c.3028 G>A/p.D1010N/NM_015102.3/3: 184910450/Heterozygous/Unknown/Variant of uncertain significance;*EHHADH*/Unknown/c.1736 G>C/p.W579S/NM_001966.3/1: 5934950/Heterozygous/Unknown/Variant of uncertain significance	41	
25	Male	71	White	Nephrology and Hypertension	Glomerulopathy	Unknown	Unknown	Uncertain Clinical Significance	*LAMA5*/Autosomal Dominant/Recessive/c.2248 G>A/p.V750M/NM_005560.3)/20: 60911471/Heterozygous/Unknown/Variant of uncertain significance	34	
26	Male	48	White	Nephrology and Hypertension	Glomerulopathy	Adulthood (31-50)	No	Positive Findings	*CYP24A1*/Autosomal Recessive/c.1396 C>T/p.R466X/NM_000782.4/20: 52773965/Heterozygous/Unknown/Pathogenic;*UPK3A*/Autosomal Dominant/c.535 C>A/p.Q179K/NM_006953.3/22: 45684988/Heterozygous/Unknown/Variant of uncertain significance	28	
27	Female	61	White	Nephrology and Hypertension	Stones	Adulthood (51-70)	Unknown	Negative Findings		40	
28	Female	45	White	Nephrology and Hypertension	CAKUT	Adulthood (18-30)	Unknown	Negative Findings		37	*HNF1A* (NM_000545.8) c.1745A>G, p.(His582Arg)
29	Male	50	African American	Nephrology and Hypertension	Glomerulopathy	Childhood	No	Uncertain Clinical Significance	*LAMA5*/Unknown/c.9211 G>A/p.D3071N/NM_005560.3/NA/Heterozygous/Unknown/Variant of uncertain significance	40	
30	Female	79	White	Nephrology and Hypertension	Stones	Unknown	Yes	Negative Findings		57	
31	Male	44	White	Nephrology and Hypertension	Glomerulopathy	Unknown	Yes	Positive Findings	*NPHS2*/Autosomal Recessive/c.948delT/p.A317LfsX31/NM_014625.3/NA/Heterozygous/Unknown/Pathogenic	40	*NPHS2* (NM_014625.4) c.686G>A; p.Arg229Gln
32	Male	22	White	Nephrology and Hypertension	CAKUT	Childhood	Yes	Uncertain Clinical Significance	*LAMC1*/Unknown/c.3845dup/p.(E1283Gfs*3)/NM_002293.3/1: 183102681/Heterozygous/Unknown/Variant of uncertain significance	37	
33	Female	21	White	Nephrology and Hypertension	Glomerulopathy	Childhood	No	Positive Findings	*NPHS2*/Autosomal Recessive/c.138_142dupGGGCT/p.S48WfsX53/NM_014625.3/1: 179544862/Heterozygous/Unknown/Pathogenic	48	
34	Female	50	Asian	Nephrology and Hypertension	Glomerulopathy	Adulthood (31-50)	Yes	Uncertain Clinical Significance	*COL4A4*/Autosomal Dominant/Recessive/c.2124 A>T/p.R708S/NM_000092.4/2: 227924892/Heterozygous/Unknown/Variant of uncertain significance	4	
35	Female	69	White	Nephrology and Hypertension	Glomerulopathy	Adulthood (51-70)	Yes	Negative Findings		37	
36	Male	78	Asian Indian	Nephrology and Hypertension	Glomerulopathy	Adulthood (51-70)	No	Uncertain Clinical Significance	*CFH*/Autosomal Dominant/Recessive/c.727 T>C/p.Y243H/NM_000186.3/1: 196648860/Heterozygous/Unknown/Variant of uncertain significance	34	
37	Male	65	White	Nephrology and Hypertension	Glomerulopathy	Unknown	Unknown	Negative Findings		31	
38	Female	47	White	Nephrology and Hypertension	Stones	Childhood	Yes	Positive Findings	*NPH1P1* / Multi-Gene/Autosomal Recessive/2q13 Deletion/Heterozygous/Father/Pathogenic	23	*RRAGD* (NM_021244.4) c.272C>T p. Thr91Ile ,
39	Female	66	White	Nephrology and Hypertension	Glomerulopathy	Unknown	Unknown	Negative Findings		31	
40	Male	70	White	Nephrology and Hypertension	Tubulointerstitial	Unknown	No	Negative Findings		26	
41	Female	43	White	Nephrology and Hypertension	CAKUT	Unknown	No	Negative Findings		20	
42	Female	71	White	Nephrology and Hypertension	Glomerulopathy	Unknown	Yes	Negative Findings		17	
43	Female	46	White	Nephrology and Hypertension	Stones	Unknown	Yes	Negative Findings		6	
44	Female	45	White	Nephrology and Hypertension	CAKUT	Adulthood (31-50)	Yes	Negative Findings		21	
45	Male	47	White	Nephrology and Hypertension	CAKUT	Unknown	No	Uncertain Clinical Significance	*CFHR5*/Autosomal Dominant/1415 C>G/S472X/NM_030787.3/1: 196973875/Heterozygous/Unknown/Variant of uncertain significance	12	
46	Female	67	White	Nephrology and Hypertension	Cysts	Unknown	Unknown	Negative Findings		42	

Supplementary Table 2: Demographic information

47	Female	63	White	Nephrology and Hypertension	CAKUT	Unknown	Unknown	Uncertain Clinical Significance	*SLC12A3*/Autosomal Recessive/c.1196_1202dupGTGATG/p.S402X/NM_000339.2/16: 56913006/Heterozygous/Unknown/Pathogenic;*SLC12A3*/Autosomal Recessive/c.434 G>A/p.R145H/NM_000339.2/16: 56902213/Heterozygous/Unknown/Variant of uncertain significance	5
48	Female	41	White	Nephrology and Hypertension	Glomerulopathy	Adulthood (31-50)	Yes	Negative Findings		56
49	Male	58	White	Nephrology and Hypertension	Tubulointerstitial	Adulthood (51-70)	Yes	Uncertain Clinical Significance	*NEK8*/Unknown/c.67 C>T/p.R23X/NM_178170.2/NA/Heterozygous/Unknown/Likely pathogenic	54
50	Male	77	African American	Nephrology and Hypertension	Cysts	Adulthood (51-70)	Yes	Uncertain Clinical Significance	*NOTCH2*/Unknown/c.6139 C>T/p.R2047W/NM_024408.3/NA/Heterozygous/Unknown/Variant of uncertain significance;*PKD2*/Unknown/c.2866 G>A/p.E956K/NM_000297.3/NA/Heterozygous/Unknown/Variant of uncertain significance	49
51	Male	67	White	Nephrology and Hypertension	Cysts	Adulthood (51-70)	Yes	Negative Findings		48
52	Female	52	White	Nephrology and Hypertension	Stones	Adulthood (18-30)	Yes	Negative Findings		3
53	Female	31	White	Nephrology and Hypertension	Glomerular (FSGS)	Childhood	No	Negative Findings		10
54	Male	70	White	Nephrology and Hypertension	Glomerulopathy	Adulthood (18-30)	No	Uncertain Clinical Significance	*PKD1*/Autosomal Dominant/c.7699A>T/p.Asn2567Tyr/NM_001009944.3/Heterozygous/Unknown/Variant of uncertain significance	27
55	Male	36	White	Nephrology and Hypertension	Glomerulopathy	Childhood	Yes	Positive Findings	*TTC21B*/Autosomal Recessive/c.626C>T/p.Pro209Leu/NM_024753.5/Homozygous/Unknown/Pathogenic	9
56	Male	14	Other	Nephrology and Hypertension	Glomerulopathy	Childhood	No	Uncertain Clinical Significance	*CUBN*/Autosomal Recessive/c.2144C>T/p.(P715L)/10:17126427/Heterozygous/Unknown/Variant of uncertain significance;*NPHS1*/Autosomal Recessive/c.2210 A>C/p.(H737P)/19:36335007/Heterozygous/Unknown/Variant of uncertain significance	11
57	Male	59	White	Nephrology and Hypertension	Glomerulopathy	Adulthood (51-70)	Yes	Negative Findings		12
58	Female	52	Other	Nephrology and Hypertension	Glomerulopathy	Adulthood (31-50)	No	Negative Findings		7
59	Female	54	White	Nephrology and Hypertension	Glomerulopathy	Adulthood (51-70)	No	Negative Findings		11
60	Male	73	Choose Not to Disclose	Pulmonary Medicine	Idiopathic Pulmonary Fibrosis	Adulthood (51-70)	Yes	Negative Findings		12
61	Female	71	White	Pulmonary Medicine	Idiopathic Pulmonary Fibrosis	Adulthood (51-70)	No	Negative Findings		22
62	Male	54	White	Pulmonary Medicine	Idiopathic Pulmonary Fibrosis	Adulthood (31-50)	No	Negative Findings		21
63	Male	63	White	Pulmonary Medicine	Idiopathic Pulmonary Fibrosis	Adulthood (31-50)	Yes	Uncertain Clinical Significance	*TERT*/Autosomal Dominant/Recessive/c.1979_1981dupCAC/p.A660_L661insP/NM_198253.2/5: 1279557/Heterozygous/Unknown/Variant of uncertain significance	13
64	Male	60	White	Pulmonary Medicine	Idiopathic Pulmonary Fibrosis	Adulthood (51-70)	No	Negative Findings		11
65	Male	77	White	Pulmonary Medicine	Idiopathic Pulmonary Fibrosis	Adulthood (>70)	Yes	Negative Findings		6
66	Male	71	White	Pulmonary Medicine	Idiopathic Pulmonary Fibrosis	Adulthood (51-70)	Yes	Negative Findings		21
67	Male	56	White	Pulmonary Medicine	Idiopathic Pulmonary Fibrosis	Adulthood (51-70)	Yes	Uncertain Clinical Significance	*ABCA3*/Autosomal Recessive/c.2125 C>T/p.(R709W)/NM_001089.2/16: 2347468/Heterozygous/Unknown/Variant of uncertain significance;*RTEL1*/Autosomal Dominant/Recessive/c.2723 C>T/p.(P908L)/NM_032957.4/20: 62323189/Heterozygous/Mother/Variant of uncertain significance;*RTEL1*/Autosomal Dominant/Recessive/c.2321 G>T/p.(R774L)/NM_032957.4/20: 62321547/Heterozygous/Mother/Variant of uncertain significance	4
68	Male	58	White	Pulmonary Medicine	Idiopathic Pulmonary Fibrosis	Adulthood (51-70)	Yes	Negative Findings		4
69	Male	74	White	Pulmonary Medicine	Idiopathic Pulmonary Fibrosis	Adulthood (>70)	Yes	Uncertain Clinical Significance	*HPS4*/Autosomal Recessive/c.1397 G>A/p.(R466H)/NM_022081.5/22: 26860199/Heterozygous/Unknown/Variant of uncertain significance	3
70	Female	82	White	Pulmonary Medicine	Idiopathic Pulmonary Fibrosis	Adulthood (>70)	Yes	Uncertain Clinical Significance	*GBA*/Autosomal Recessive/c.1279 G>A/p.(E427K)/NM_001005741.2/1: 155205581/Heterozygous/Unknown/Variant of uncertain significance;*SLC34A2*/Autosomal Recessive/c.1207 A>G/p.(I403V)/NM_006424.2/4: 25674867/Heterozygous/Unknown/Variant of uncertain significance	4
71	Female	79	White	Pulmonary Medicine	Idiopathic Pulmonary Fibrosis	Adulthood (51-70)	No	Negative Findings		41

Supplementary Table 2: Demographic information

72	Male	14	White	Rheumatology	Auto-inflammatory Syndromes	Childhood	No	Negative Findings		21
73	Female	20	White	Rheumatology	Auto-inflammatory Syndromes	Childhood	No	Uncertain Clinical Significance	*NOD2*/Autosomal Dominant/c.566 C>T/p.T189M/NM_022162.1/16: 50741791/Heterozygous/Mother/Variant of uncertain significance;*TMEM173*/Autosomal Dominant/c.1109 A>G/p.K370R/NM_198282.3/5: 138855877/Heterozygous/Mother/Variant of uncertain significance	21
74	Female	6	White	Rheumatology	Auto-inflammatory Syndromes	Childhood	No	Uncertain Clinical Significance	*ITK*/Autosomal Recessive/c.1551 C>T/p.G517=/NM_005546.3/5: 156672927/Heterozygous/Mother/Variant of uncertain significance	20
75	Female	6	White	Rheumatology	Auto-inflammatory Syndromes	Childhood	Yes	Uncertain Clinical Significance	*PSTPIP1*/Autosomal Dominant/c.566 G>A/p.R189Q/NM_003978.3/15: 77322846/Heterozygous/Father/Variant of uncertain significance	16
76	Female	21	White	Rheumatology	Auto-inflammatory Syndromes	Childhood	Yes	Uncertain Clinical Significance	*BTK*/X-linked/c.500 T>A/p.I167N/NM_000061.2/X: 100617569/Heterozygous/Unknown/Variant of uncertain significance	33
77	Female	19	White	Rheumatology	Auto-inflammatory Syndromes	Childhood	Yes	Uncertain Clinical Significance	*PRF1*/Autosomal Recessive/c.272 C>T/p.A91V/NM_001083116.1/NA/Heterozygous/Unknown/Risk allele	15
78	Male	11	White	Rheumatology	Auto-inflammatory Syndromes	Childhood	Yes	Uncertain Clinical Significance	*NLRP1*/Autosomal Dominant/Recessive/c.3973 G>A/p.V1325I/NM_033004.3/17: 5421150/Heterozygous/Mother/Variant of uncertain significance	16
79	Female	24	White	Rheumatology	Auto-inflammatory Syndromes	Adulthood (18-30)	No	Uncertain Clinical Significance	*G6PC3*/Autosomal Recessive/c.596 A>T/p.Y199F/NM_138387.3/17: 42152738/Heterozygous/Mother/Variant of uncertain significance;*MEFV*/Autosomal Recessive/c.442 G>C/p.E148Q/NM_000243.2/16: 3304626/Heterozygous/Mother/Variant of uncertain significance;*NOD2*/Autosomal Dominant/c.140 C>T/p.S47L/NM_022162.1/16: 50733465/Heterozygous/Mother/Variant of uncertain significance;*PLCG2*/Autosomal Dominant/c.2312 A>G/p.Q771R/NM_002661.4/16: 81957094/Heterozygous/Father/Variant of uncertain significance;*PSTPIP1*/Autosomal Dominant/c.839 C>T/p.A280V/NM_003978.3/15: 77325203/Heterozygous/Father/Variant of uncertain significance;*DNASE1*/Unknown/c.319 A>G/p.R107G/NM_005223.3/16: 3706185/Heterozygous/Father/Variant of uncertain significance	13
80	Female	15	White	Rheumatology	Auto-inflammatory Syndromes	Childhood	No	Uncertain Clinical Significance	*DCLRE1C*/Autosomal Recessive/c.799 T>C/p.W267R/NM_001033855.1/10: 14970133/Heterozygous/Father/Variant of uncertain significance;*ELANE*/Autosomal Dominant/c.740 A>T/p.Q247L/NM_001972.2/19: 856100/Heterozygous/Father/Variant of uncertain significance;*NLRP3*/Autosomal Dominant/c.598 G>A/p.V200M/NM_004895.4/1: 247587343/Heterozygous/Father/Variant of uncertain significance;*PSMB9*/Unknown/c.517 C>T/p.R173C/NM_002800.4/6: 32826267/Heterozygous/Mother/Variant of uncertain significance	12
81	Male	5	White	Rheumatology	Auto-inflammatory Syndromes	Childhood	No	Uncertain Clinical Significance	*CARD14*/Autosomal Dominant/c.1082_1089+27del35/Heterozygous/Mother/Variant of uncertain significance;*RAG2*/Autosomal Recessive/c.1222 G>A/p.E408K/Heterozygous/Mother/Variant of uncertain significance;*TNFAIP3*/Autosomal Dominant/c.2231 G>A/p.G744D/Heterozygous/Mother/Variant of uncertain significance	27
82	Female	5	White	Rheumatology	Auto-inflammatory Syndromes	Childhood	No	Uncertain Clinical Significance	*LIG4*/Autosomal Recessive/c.108C>G/P.I36M/NM_002312.3/13: 108863509/Heterozygous/Mother/Variant of uncertain significance	6
83	Male	20	White	Rheumatology	Auto-inflammatory Syndromes	Childhood	No	Uncertain Clinical Significance	*STAT1*/Autosomal Dominant/Recessive/c.1098-11T>A/p.?/NM_007315.3/2: 191851805/Heterozygous/Father/Variant of uncertain significance	10
84	Female	51	White	Rheumatology	Auto-inflammatory Syndromes	Adulthood (31-50)	Yes	Uncertain Clinical Significance	*TRNT1*/Autosomal Recessive/c.809 C>G/p.P270R/MN_182916.2/3: 3189140/Heterozygous/Unknown/Variant of uncertain significance	7
85	Male	15	White	Rheumatology	Auto-inflammatory Syndromes	Childhood	Yes	Uncertain Clinical Significance	ADA/Autosomal Recessive/c.591T>A/p.H197Q/NM_000022.2/20: 43252858/Heterozygous/Father/Variant of uncertain significance	7
86	Female	19	White	Rheumatology	Auto-inflammatory Syndromes	Childhood	Yes	Uncertain Clinical Significance	*AP3B1*/Autosomal Recessive/c.1198 G>C/p.(A400P)/NM_003664.3/5: 77461466/Heterozygous/Father/Variant of uncertain significance;*DOCK8*/Autosomal Recessive/c.4774 T>C/p.(F1592L)/NM_203447.3/9: 432313/Heterozygous/Father/Variant of uncertain significance	7
87	Female	34	White	Rheumatology	Auto-inflammatory Syndromes	Adulthood (31-50)	No	Uncertain Clinical Significance	*TNFAIP3*/Autosomal Dominant/c.2231 G>A/p.(G744D)/NM_006290.3/6: 138202314/Heterozygous/Unknown/Variant of uncertain significance	5
88	Female	12	White	Rheumatology	Auto-inflammatory Syndromes	Childhood	Yes	Negative Findings		3
89	Female	39	White	Rheumatology	Auto-inflammatory Syndromes	Adulthood (31-50)	Yes	Uncertain Clinical Significance	*ELANE*/Autosomal Dominant/c.740 A>T/p.(Q247L)/NM_001972.2/19: 856100/Heterozygous/Unknown/Variant of uncertain significance;PIK3CD/Autosomal Dominant/c.854 T>C/p.(M285T)/NM_005026.3/1: 9777090/Heterozygous/Unknown/Variant of uncertain significance;*PIK3R1*/Autosomal Dominant/Recessive/c.586 C>T/p.(P196S)/NM_181523.2/5: 67575513/Heterozygous/Unknown/Variant of uncertain significance;*RAG2*/Autosomal Recessive/c.368 G>A/p.(R123H)/NM_000536.3/11: 36615351/Heterozygous/Unknown/Variant of uncertain significance;*RNASEH2B*/Autosomal Recessive/c.787 A>G/p.(T263A)/NM_024570.3/13: 51528086/Heterozygous/Unknown/Variant of uncertain significance	3
90	Female	40	White	Rheumatology	Auto-inflammatory Syndromes	Adulthood (18-30)	Yes	Uncertain Clinical Significance	*PRF1*/Autosomal Recessive/c.272 C>T/p.(A91V)/NM_001083116.1/NA/Heterozygous/Unknown/Risk allele	7
91	Female	26	White	Rheumatology	Auto-inflammatory Syndromes	Childhood	Unknown	Uncertain Clinical Significance	*PRF1*/Autosomal Recessive/c.272 C>T/p.(A91V)/NM_001083116.1/10: 72360387/Heterozygous/Unknown/Risk allele;*PSMB9*/Unknown/c.517	2

Supplementary Table 2: Demographic information

									C>T/p.(R173C)/NM_002800.4/6: 32826267/Heterozygous/Unknown/Variant of uncertain significance	
92	Female	32	White	Rheumatology	Auto-inflammatory Syndromes	Adulthood (18-30)	No	Negative Findings		1
93	Female	67	White	Rheumatology	Auto-inflammatory Syndromes	Childhood	Yes	Uncertain Clinical Significance	*SLC37A4*/Autosomal Recessive/c.857 G>A/p.(R286Q)/NM_001164277.1/11: 118897326/Heterozygous/Unknown/Variant of uncertain significance;*WAS*/X-linked/c.1112 C>T/p.(P371L)/NM_000377.2)/X: 48547229/Heterozygous/Unknown/Variant of uncertain significance	9
94	Male	32	White	Rheumatology	Auto-inflammatory Syndromes	Adulthood (31-50)	Yes	Uncertain Clinical Significance	*NLRP12*/Autosomal Dominant/c.586A>G/p.Ile196Val/NM_144687.4/Heterozygous/Unknown/Variant of uncertain significance	9
95	Female	38	White	Rheumatology	Auto-inflammatory Syndromes	Adulthood (31-50)	Yes	Negative Findings		10
96	Male	19	White	Rheumatology	Auto-inflammatory Syndromes	Childhood	No	Negative Findings		6
97	Female	43	White	Rheumatology	Auto-inflammatory Syndromes	Adulthood (31-50)	Yes	Positive Findings	*NOD2*/Autosomal Dominant/c.2023C>T/p.Arg675Trp/NM_001370466.1/Heterozygous/Unknown;NOD2/Autosomal Dominant/c.2717+158C>T/NM_001370466.1/Heterozygous/Unknown	8
98	Female	40	White	Rheumatology	Auto-inflammatory Syndromes	Childhood	Yes	Negative Findings		7
99	Male	21	White	Rheumatology	Auto-inflammatory Syndromes	Childhood	Yes	Negative Findings		6
100	Female	42	White	Rheumatology	Auto-inflammatory Syndromes	Adulthood (31-50)	Unknown	Negative Findings		2
